# Species Diversity of *Cordyceps*-Like Fungi in the Tiankeng Karst Region of China

**DOI:** 10.1128/spectrum.01975-22

**Published:** 2022-09-12

**Authors:** Wan-Hao Chen, Jian-Dong Liang, Xiu-Xiu Ren, Jie-Hong Zhao, Yan-Feng Han, Zong-Qi Liang

**Affiliations:** a Center for Mycomedicine Research, Basic Medical School, Guizhou University of Traditional Chinese Medicine, Guiyang, Guizhou, People’s Republic of China; b Institute of Fungus Resources, Department of Ecology, College of Life Sciences, Guizhou University, Guiyang, Guizhou, People’s Republic of China; The Ohio State University

**Keywords:** Tiankeng, ascomycete, *Cordyceps*-like fungi, phylogenetic analysis, morphological

## Abstract

Tiankeng acts as a refugium for biodiversity amid a changing global climate, and a previous study has shown that some ancient (*Alsophila spinulosa*) and unique plants (cool-adapted plants) are present in Tiankeng. However, there are few reports on Ascomycota from the Tiankeng karst region. In this research, the species diversity of *Cordyceps*-like fungi in Monkey-Ear Tiankeng was investigated. Seven species in the genera *Akanthomyces*, *Beauveria*, *Cordyceps*, and *Samsoniella* were identified based on internal transcribed spacer sequences and morphological characteristics. Eight new species in the genera *Akanthomyces*, *Cordyceps*, and *Samsoniella* were established and described according to a multilocus phylogenetic analysis and morphological characteristics. Our results revealed that *Cordyceps*-like fungi were abundant in Monkey-Ear Tiankeng, providing new insights into the diversity of Ascomycota in this special eco-environment.

**IMPORTANCE** Karst Tiankeng has a special eco-environment and acts as a refugium for biodiversity. However, there are few reports on Ascomycota from the Tiankeng karst region. In this research, seven known species and eight new species in the genera *Akanthomyces*, *Beauveria*, *Cordyceps*, and *Samsoniella* were reported. The results showed that *Cordyceps*-like fungi are abundant in Monkey-Ear Tiankeng. Interestingly, the month of the sampling was November, which is not an active period of growth and reproduction for *Cordyceps*-like fungi. These results revealed that unconventional time sampling should not be ignored, especially for a special eco-environment, and provided new insights into the diversity of Ascomycota in this special eco-environment.

## INTRODUCTION

Tiankeng is a kind of negative karst terrain, which was first named by Zhu in 2001 (1), and it has developed from a carbonate rock stratum, is connected with an underground river at the bottom, and is surrounded by steep rock walls and an aquifer vadose zone with a continuous sedimentary thickness (2). The unique geological landform of Tiankeng creates a microclimate that is different from those of its surrounding areas and acts as a haven of biodiversity in the context of changing climates (3–5).

Research on the Tiankeng karst region has been conducted for many years and has focused on geology (including the morphology, formation, and evolution mechanism of Tiankeng) (6–10), animals (11, 12), plants (13–15), soil microbiology (16–18), the value of Tiankeng's tourism resources (19), and the settlement of organic pollutants in Tiankeng (20, 21). A high floristic diversity, abundant species, a remarkably high uniqueness, a water-bearing capability, and endemic species have been found in the Tiankeng karst region (4). In particular, there is a large amount of water and nutrients present at the bottom of the Tiankeng karst region, which strongly affects the composition and structure of the vegetation (22, 23).

Jiang et al. (24) noted that the soil fungus *Trichoderma* sp. was able to degrade feathers efficiently. Lan et al. (25) reported that the fungus *Exophiala* sp., which was isolated from the soil of the Tiankeng karst region, was able to improve the drought resistance and growth of *Zenia insignis* Chun and *Caesalpinia sappan* Linn. Long et al. (18) reported a new species, *Tiankengomelania guangxiense*, which was shown to be able to promote the growth of the medicinal orchid *Dendrobium officinale* Kimura et Migo and was isolated from the rhizosphere soils of a virgin forest in the Baidong Tiankeng. However, there are few reports regarding the *Cordyceps*-like fungi isolated from the Tiankeng karst region.

During a survey of entomopathogenic fungi from Southwest China, some *Cordyceps*-like fungi were found in Monkey-Ear Tiankeng. Eight new species distributed across the three genera *Akanthomyces*, *Cordyceps*, and *Samsoniella* were established based on a multilocus phylogeny as well as their morphological and ecological characteristics.

## RESULTS

### Phylogenetic analyses.

**FIG 1:**
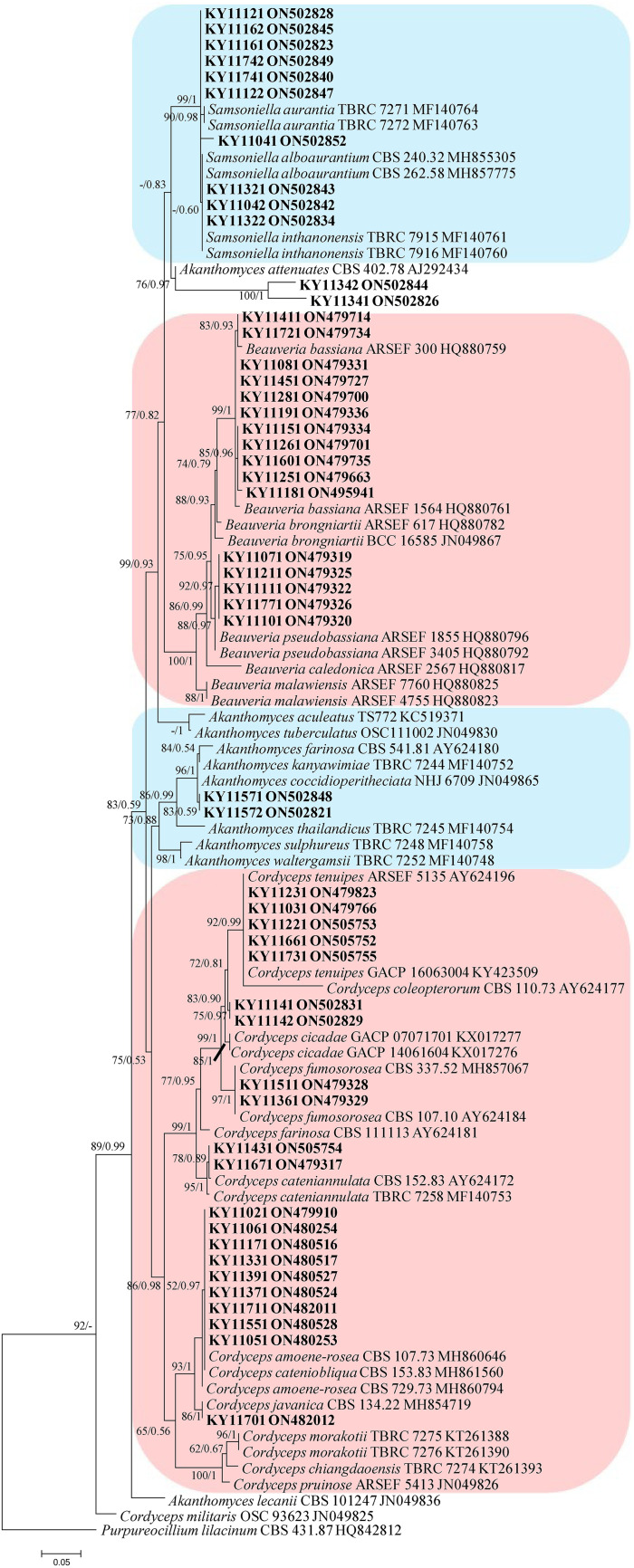
Phylogenetic relationships among the new strains and their allies based on an internal transcribed spacer (ITS) sequence. Statistical support values (≥0.5/50%) are shown at the nodes for maximum likelihood (ML) bootstrap support/Bayesian inference (BI) posterior probabilities.

**FIG 2:**
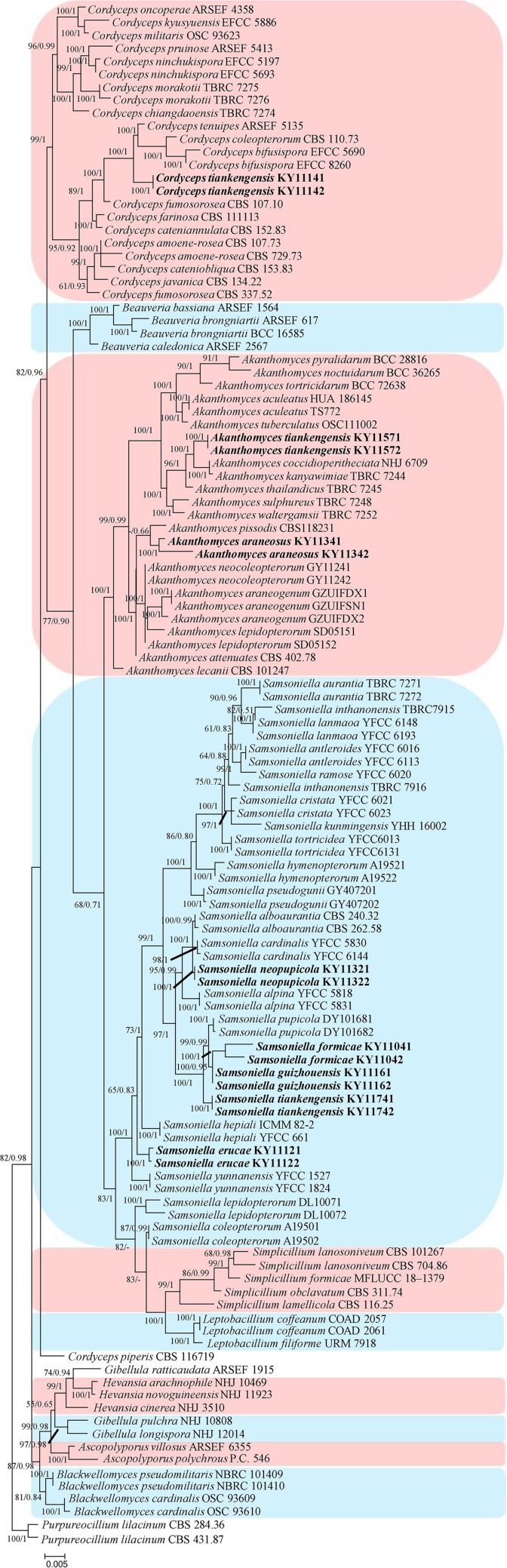
Phylogenetic relationships among the new strains and their allies based on a multilocus data set (ITS, large subunit rRNA [LSU], RNA polymerase II largest subunit 2 [RPB2], and translation elongation factor 1 alpha [TEF]). Statistical support values (≥0.5/50%) are shown at the nodes for ML bootstrap support/BI posterior probabilities.

In the phylogenetic tree of analysis 1 (establishing the genus placement of the new strains) and analysis 2 (determining the establishment of the new species) (Fig. 1 and 2, respectively), Purpureocillium lilacinum (Thom) Luangsa-ard, Houbraken, and Hywel-Jones & Samson (CBS 431.87) were used as the outgroup in analysis 1, whereas *P. lilacinum* (CBS 284.36 and CBS 431.87) was used as the outgroup in analysis 2. The concatenated sequences of analyses 1 and 2 included 32 and 74 taxa, respectively, and consisted of 516 (internal transcribed spacer [ITS], 516) and 2,480 (ITS, 540; large subunit rRNA [LSU], 586; RNA polymerase II largest subunit 2 [RPB2], 671; and translation elongation factor 1 alpha [TEF], 683) characters with gaps, respectively.

Analysis 1: The final value of the highest scoring tree was –3,525.3844, which was obtained from a maximum likelihood (ML) analysis of ITS sequence. The parameters of the general time-reversible (GTR) model used to analyze the data set were estimated using the following frequencies: A = 0.2280, C = 0.3330, G = 0.2552, and T = 0.1838 with the substitution rates AC = 1.9959, AG = 2.6768, AT = 1.0000, CG = 1.9959, CT = 6.7579 and GT = 1.0000. The gamma distribution shape parameter was α = 0.4764. The selected model for the Bayesian inference (BI) analysis was GTR+F+G4 (ITS). The phylogenetic trees (Fig. 1) constructed using the ML and BI analyses were largely congruent and strongly supported in most branches. The new strains were clustered into to the following genera: *Akanthomyces* Lebert, *Cordyceps* Fr., *Beauveria* Vuill., and *Samsoniella* Mongkols., Noisrip., Thanakitp., Spatafora & Luangsa-ard species, respectively. Strains KY11411, KY11721, KY11081, KY11451, KY11281, KY11191, KY11151, KY11261, KY11601, KY11251, and KY11181 were clustered into the subclade of Beauveria bassiana (Bals.-Criv.) Vuill. with a high bootstrap value (99/1). Strains KY11071, KY11211, KY11111, KY11771, and KY11101 were clustered into the subclade of *B. pseudobassiana* S.A. Rehner & Humber with a high bootstrap value (92/0.97). Strains KY11231, KY11031, KY11221, KY11661, and KY11731 were clustered into the subclade of *Cordyceps tenuipes* (Peck) Kepler, B. Shrestha & Spatafora with a high bootstrap value (92/0.99). Strains KY11511 and KY11361 were clustered into the subclade of *C. fumosorosea* (Wize) Kepler, B. Shrestha & Spatafora with a high bootstrap value (99/1). Strains KY11431 and KY11671 were clustered into the subclade of *C. cateniannulata* (Z.Q. Liang) Kepler, B. Shrestha & Spatafora with a high bootstrap value (95/1). Strains KY11021, KY11061, KY11171, KY11331, KY11391, KY11371, KY11711, KY11551, and KY11051 were clustered with *C. amoene-rosea* (Henn.) Kepler, B. Shrestha & Spatafora and *C. cateniobliqua* (Z.Q. Liang) Kepler, B. Shrestha & Spatafora. Strain KY11701 was clustered into the subclade of *C. javanica* (Bally) Kepler, B. Shrestha & Spatafora with a high bootstrap value (86/1). Strains KY11121, KY11122, KY11161, KY11162, KY11741, KY11742, KY11041, KY11042, KY11321 ,and KY11322 were clustered into the clade of the genus *Samsoniella* Mongkols., Noisrip., Thanakitp., Spatafora & Luangsa-ard. Strains KY11341, KY11342, KY11571, and KY11572 were all clustered with the *Akanthomyces* species. Strains KY11141 and KY11142 were clustered into the clade of the genus *Cordyceps*.

Comparing their typical morphological characteristics, strains KY11411, KY11721, KY11081, KY11451, KY11281, KY11191, KY11151, KY11261, KY11601, KY11251, and KY11181 were identified as Beauveria bassiana. Strains KY11071, KY11211, KY11111, KY11771, and KY11101 were identified as *B. pseudobassiana*. Strains KY11231, KY11031, KY11221, KY11661, and KY11731 were identified as *Cordyceps tenuipes*. Strains KY11511 and KY11361 were identified as *C. fumosorosea*. Strains KY11431 and KY11671 were identified as *C. cateniannulata*. Strains KY11021, KY11061, KY11171, KY11331, KY11391, KY11371, KY11711, KY11551, and KY11051 were identified as *C. cateniobliqua*. Strain KY11701 was identified as *C. javanica*. Multilocus phylogenetic analysis was required for the further identification of the strains KY11121, KY11122, KY11161, KY11162, KY11741, KY11742, KY11041, KY11042, KY11321, KY11322, KY11341, KY11342, KY11571, KY11572, KY11141, and KY11142.

Analysis 2: The final value of the highest scoring tree was −28,566.0385, which was obtained from the ML analysis of the data set (ITS+LSU+RPB2+TEF). The parameters of the GTR model used to analyze the data set were estimated based on the following frequencies: A = 0.2351, C = 0.2835, G = 0.2710, and T = 0.2104 with the substitution rates AC = 1.0000, AG = 2.2438, AT = 1.0000, CG = 1.0000, CT = 5.0707, and GT = 1.0000. The gamma distribution shape parameter was α = 0.5592. The selected models for the BI analysis were GTR+F+I+G4 (ITS, TEF), GTR+F+G4 (LSU), and SYM+G4 (RPB2). The phylogenetic trees (Fig. 2) constructed using the ML and BI analyses were largely congruent and strongly supported in most branches. Most of the genera were clustered into their independent clades. Strains KY11141 and KY11142 were clustered with *Cordyceps tenuipes* (Peck) Kepler, B. Shrestha & Spatafora, *C. coleopterorum* (Samson & H.C. Evans) Kepler, B. Shrestha & Spatafora, and *C. bifusispora* O.E. Erikss. in a subclade. Strains KY11571 and KY11572 were clustered with *Akanthomyces coccidioperitheciata* (Kobayasi & Shimizu) Spatafora, Kepler & B. Shrestha, *A. kanyawimiae* Mongkols., Noisrip., Thanakitp., Spatafora & Luangsa-ard, and *A. thailandicus* Mongkols., Spatafora & Luangsa-ard in a subclade. Strains KY11341 and KY11342 were clustered with *A. pissodis* (Kope & I. Leal) W.H. Chen, Y.F. Han & Z.Q. Liang in a subclade. Strains KY11321 and KY11322 were clustered with *Samsoniella alboaurantia* (G. Sm.) Mongkols., Noisrip., Thanakitp., Spatafora & Luangsa-ard and *S. cardinalis* H. Yu, Y.B. Wang, Y. Wang, Q. Fan & Zhu L. Yang in a subclade. Strains KY11041, KY11042, KY11161, and KY11162 were clustered with *S. pupicola* W.H. Chen, Y.F. Han, J.D. Liang & Z.Q. Liang in a subclade. Strains KY11741, KY11742, KY11121, and KY11122 were clustered into two independent clades.

**FIG 3:**
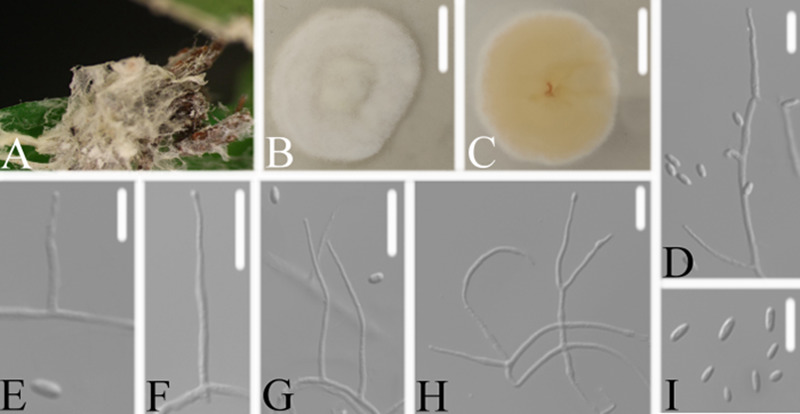
*Akanthomyces araneosus*. (A) Infected spider (Araneae). (B, C) PDA-containing culture viewed from above (B) and below (C). (D–I) Phialides and conidia. Scale bars: 10 mm (B, C) and 10 μm (D–I).

Eight new species distributed in the three genera *Akanthomyces, Cordyceps* and *Samsoniella*, were established based on a multi-locus phylogeny, their morphological and ecological characteristics. The morphological and ecological characteristics of the new species were as follows. *Akanthomyces araneosus* W.H. Chen, Y.F. Han, J.D. Liang & Z.Q. Liang, sp. nov (MycoBank: 844985) (Fig. 3).

Type: China, Guizhou, Guiyang, Kaiyang County, Monkey-Ear Tiankeng (27°5'12.138'' N, 107°0'48.42'' E). On a dead spider (Araneae), 16 November 2020, Wanhao Chen, GZAC KY1134 (holotype), ex-type living cultures, KY11341.

Description: Spider host was completely covered by white mycelium. Conidiophores were mononematous and arose from the lateral hyphae. Colonies on potato dextrose agar (PDA) were 2.4 to 2.6 cm in diameter after 14 d at 25°C, white, and comprised of a basal felt and a floccose hyphal overgrowth with the reverse yellowish. Prostrate hyphae were smooth, septate, hyaline, and 1.0 to 2.5 μm in diameter. Erect conidiophores usually arose from the aerial hyphae. Phialides were solitary or in groups of two. Phialides were 16.9 to 18.1 × 1.3 to 1.9 μm with a cylindrical basal portion and tapered into a short, distinct neck. Conidia were hyaline, fusiform, one-celled, and 3.1 to 5.0 × 1.0 to 1.8 μm. The sexual state was not observed.

Host: Spider (Araneae).

Locality: Kaiyang County (27°5'12.138'' N, 107°0'48.42'' E), Guiyang, Guizhou Province, China.

Etymology: Referring to the mycelium covering the spider like a spider web.

Additional strain examined: China, Guizhou, Guiyang, Kaiyang County (27°5'12.138'' N, 107°0'48.42'' E). On a dead spider (Araneae), 16 November 2020, Wanhao Chen, KY11342.

Remarks: *Akanthomyces araneosus* was easily identified as *Akanthomyces*, according to the phylogenetic analysis of the combined data sets (ITS, LSU, RPB2, TEF) (Fig. 1 and 2), and has a close relationship with *A. pissodis*. When comparing the typical characteristics, *A. araneosus* was easily distinguished from *A. pissodis* by its fusiform, smaller conidia (3.1 to 5.0 × 1.0 to 1.8 μm) and its spider host.

**FIG 4:**
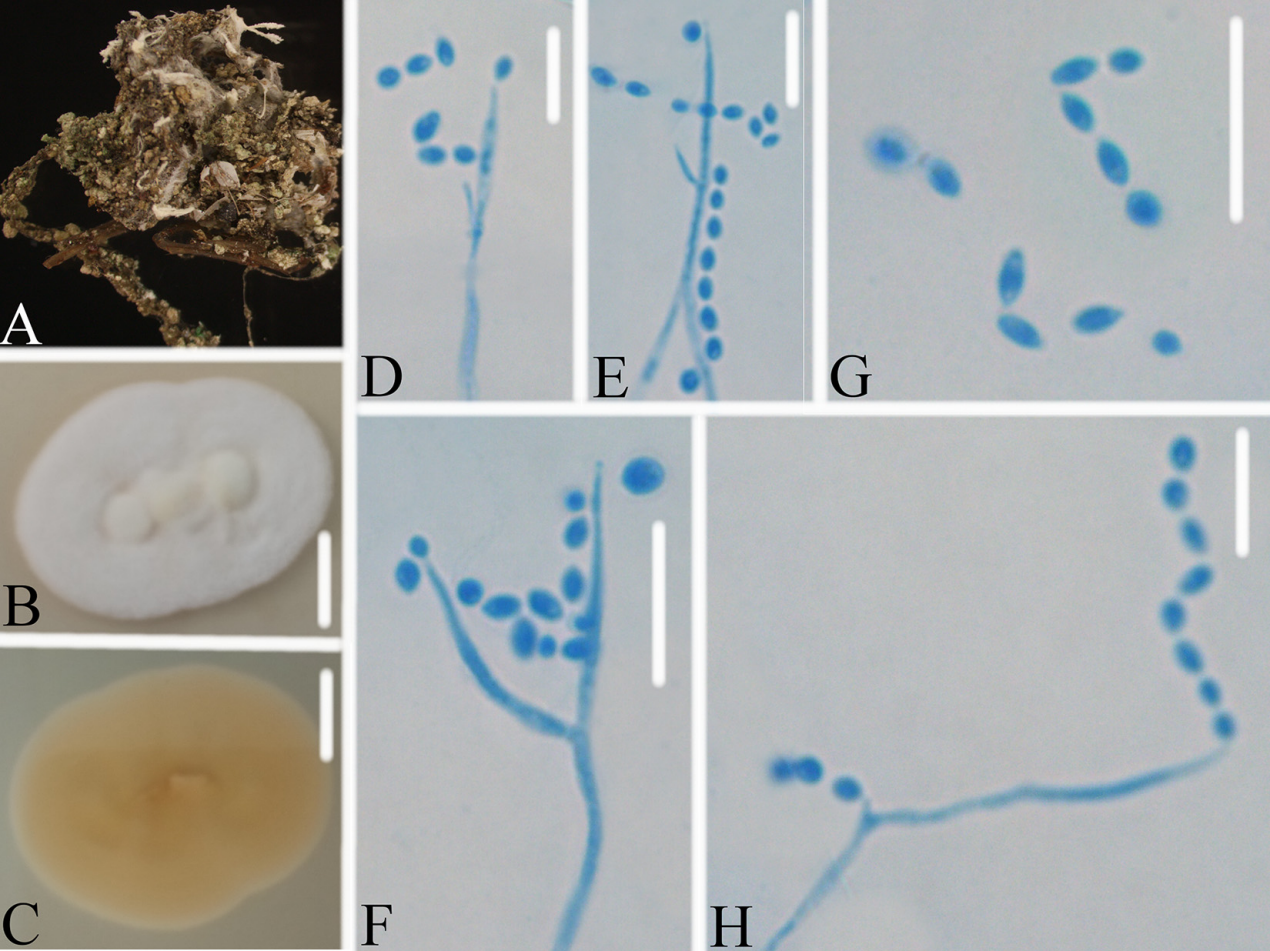
*Akanthomyces tiankengensis*. (A) Infected spider (Araneae). (B, C) PDA-containing culture viewed from above (B) and below (C). (D–H) Phialides and conidia. Scale bars: 10 mm (B, C) and 10 μm (D–H).

*Akanthomyces tiankengensis* W.H. Chen, Y.F. Han, J.D. Liang & Z.Q. Liang, sp. nov. (MycoBank: 844986) (Fig. 4).

Type: China, Guizhou, Guiyang, Kaiyang County, Monkey-Ear Tiankeng (27°5'12.138'' N, 107°0'48.42'' E). On a dead spider (Araneae), 16 November 2020, Wanhao Chen, GZAC KY1157 (holotype), ex-type living cultures, KY11571.

Description: Spider host was completely covered by white mycelium. Conidiophores were mononematous and arose from the lateral hyphae. Colonies on PDA were 2.8 to 3.6 cm in diameter after 14 d at 25°C, white, and comprised of a basal felt and a floccose hyphal overgrowth with the reverse yellowish. Prostrate hyphae were smooth, septate, hyaline, and 1.2 to 2.9 μm in diameter. Erect conidiophores usually arose from the aerial hyphae. Phialides were solitary or in groups of two. Phialides were 13.9 to 17.1 × 1.1 to 1.6 μm with a cylindrical basal portion and tapered into a short, distinct neck. Conidia were hyaline, fusiform, one-celled, and 2.3 to 3.0 × 1.5 to 2.3 μm. The sexual state was not observed.

Host: Spider (Araneae).

Locality: Kaiyang County (27°5'12.138” N, 107°0'48.42” E), Guiyang, Guizhou Province, China.

Etymology: Referring to its location in Tiankeng.

Additional strain examined: China, Guizhou, Guiyang, Kaiyang County (27°5'12.138” N, 107°0'48.42” E). On a dead spider (Araneae), 16 November 2020, Wanhao Chen, KY11572.

Remarks: *Akanthomyces tiankengensis* was easily identified as *Akanthomyces*, according to the phylogenetic analysis of the combined data sets (ITS, LSU, RPB2, TEF) (Fig. 1 and 2), and has a close relationship with *A. coccidioperitheciata*, *A. kanyawimiae*, and *A. thailandicus*. When comparing the typical characteristics, *A. tiankengensis* was easily distinguished from *A. coccidioperitheciata* by the absence of a teleomorph and the presence of a spider host. It was distinguished from *A. kanyawimiae* by its longer phialides (13.9 to 17.1 × 1.1 to 1.6 μm) and fusiform conidia. It was distinguished from *A. thailandicus* by its smaller conidia (2.3 to 3.0 × 1.5 to 2.3 μm) and cylindrical phialides.

**FIG 5:**
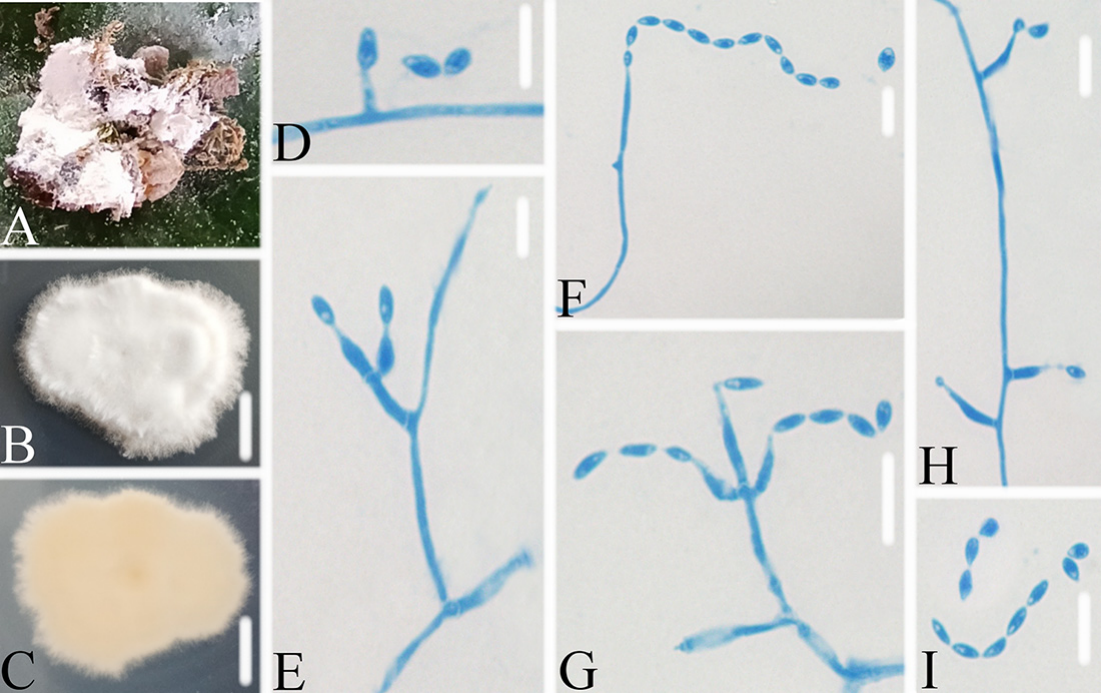
*Cordyceps tiankengensis*. (A) Infected pupa (Lepidoptera). (B, C) PDA-containing culture viewed from above (B) and below (C). (D–I). Phialides and conidia. Scale bars: 10 mm (B, C) and 10 μm (D–I).

*Cordyceps tiankengensis* W.H. Chen, Y.F. Han, J.D. Liang & Z.Q. Liang, sp. nov. (MycoBank: 844987) (Fig. 5).

Type: China, Guizhou, Guiyang, Kaiyang County, Monkey-Ear Tiankeng (27°5'12.138” N, 107°0'48.42” E). On a pupa (Lepidoptera), 16 November 2020, Wanhao Chen, GZAC KY1114 (holotype), ex-type living cultures, KY11141.

Description: Insect pupa was completely covered by white mycelium. Conidiophores were mononematous and arose from the lateral hyphae. Colonies on PDA were 2.2 to 3.2 cm in diameter after 14 d at 25°C, white, and comprised of a basal felt and a floccose hyphal overgrowth with the reverse yellowish. Prostrate hyphae were smooth, septate, hyaline, and 1.3 to 1.9 μm in diameter. Erect conidiophores usually arose from the aerial hyphae. Phialides were solitary or in groups of two. Phialides were 7.1 to 12.2 × 1.9 to 2.5 μm with a cylindrical basal portion and tapered into a short, distinct neck. Conidia were in chains, hyaline, fusiform, one-celled, and 4.1 to 5.1 × 1.8 to 2.7 μm. The sexual state was not observed.

Host: Pupa (Lepidoptera).

Locality: Kaiyang County (27°5'12.138” N, 107°0'48.42” E), Guiyang, Guizhou Province, China.

Etymology: Referring to its location in Tiankeng.

Additional strain examined: China, Guizhou, Guiyang, Kaiyang County (27°5'12.138” N, 107°0'48.42” E). On a pupa (Lepidoptera), 16 November 2020, Wanhao Chen, KY11142.

Remarks: *Cordyceps tiankengensis* was easily identified as *Cordyceps*, according to the phylogenetic analysis of combined data sets (ITS, LSU, RPB2, TEF) (Fig. 1 and 2), and has a close relationship with *C. tenuipes* and *C. coleopterorum*. When comparing the typical characteristics, *C. tiankengensis* was easily distinguished from *C. tenuipes* by its longer phialides (7.1 to 12.2 × 1.9 to 2.5 μm), and it was easily distinguished from *C. coleopterorum* by its smaller conidia (4.1 to 5.1 × 1.8 to 2.7 μm).

**FIG 6:**
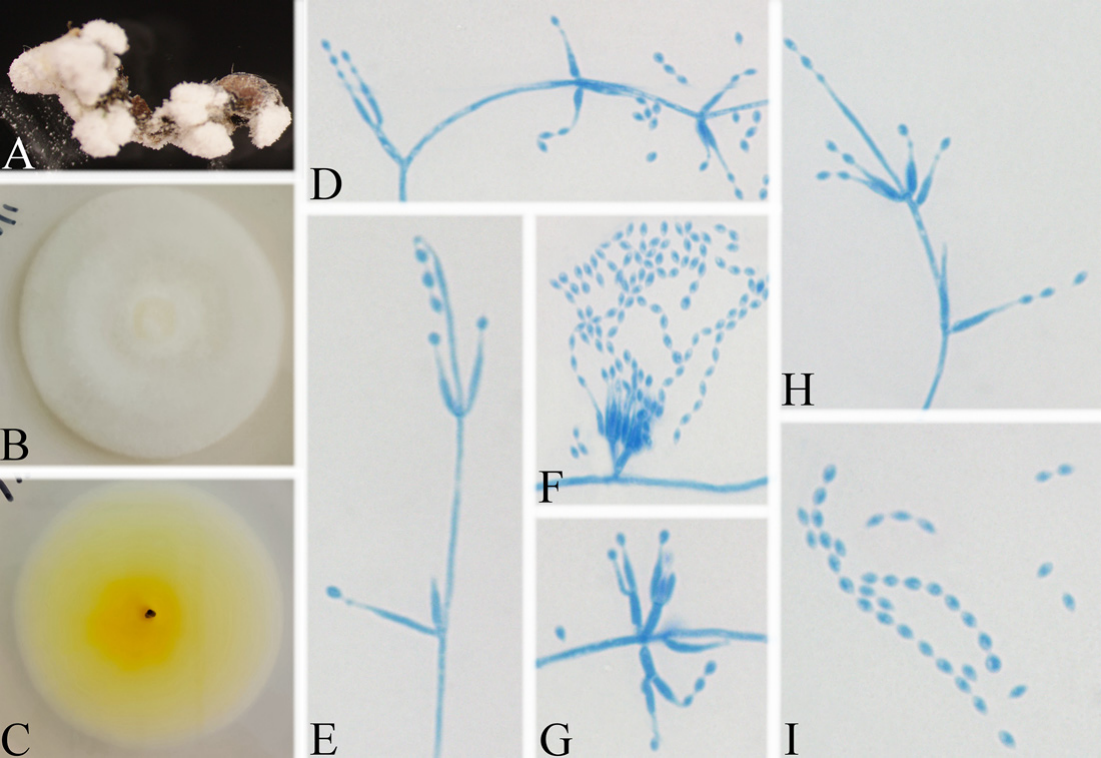
*Samsoniella formicae*. (A) Infected ant (Formicidae). (B, C) PDA-containing culture viewed from above (B) and below (C). (D–I) Phialides and conidia. Scale bars: 10 mm (B, C) and 10 μm (D–I).

*Samsoniella formicae* W.H. Chen, Y.F. Han, J.D. Liang & Z.Q. Liang, sp. nov. (MycoBank: 844988) (Fig. 6).

Type: China, Guizhou, Guiyang, Kaiyang County, Monkey-Ear Tiankeng (27°5'12.138” N, 107°0'48.42” E). On an ant (Formicidae), 16 November 2020, Wanhao Chen, GZAC KY1104 (holotype), ex-type living cultures, KY11041.

Description: Ant host was completely covered by white mycelium. Conidiophores were mononematous and arose from the lateral hyphae. Colonies on PDA were 5.0 to 5.1 cm in diameter after 14 d at 25°C, white, and comprised of a basal felt and a floccose hyphal overgrowth with the reverse yellowish or pale orange. Prostrate hyphae were smooth, septate, hyaline, and 1.3 to 1.9 μm in diameter. Erect conidiophores usually arose from the aerial hyphae. Phialides were solitary or in groups of two. Phialides were 8.3 to 13.5 × 1.2 to 1.8 μm with a cylindrical basal portion and tapered into a short, distinct neck. Conidia were in chains, hyaline, fusiform, one-celled, and 2.5 to 3.2 × 1.6 to 2.2 μm. The sexual state was not observed.

Host: Ant (Formicidae).

Locality: Kaiyang County (27°5'12.138” N, 107°0'48.42” E), Guiyang, Guizhou Province, China.

Etymology: Referring to its ant host.

Additional strain examined: China, Guizhou, Guiyang, Kaiyang County (27°5'12.138” N, 107°0'48.42” E). On an ant (Formicidae), 16 November 2020, Wanhao Chen, KY11042.

Remarks: *Samsoniella formicae* was easily identified as *Samsoniella*, according to the phylogenetic analysis of the combined data sets (ITS, LSU, RPB2, TEF) (Fig. 1 and 2), and has a close relationship with *S. pupicola* and *S. guzhouensis*. When comparing the typical characteristics, *S. formicae* was easily distinguished from *S. pupicola* by its longer phialides (8.3 to 13.5 × 1.2 to 1.8 μm) and its ant host. It was distinguished from *S. guzhouensis* by its longer phialides (8.3 to 13.5 × 1.2 to 1.8 μm) and its bigger conidia (2.5 to 3.2 × 1.6 to 2.2 μm).

**FIG 7:**
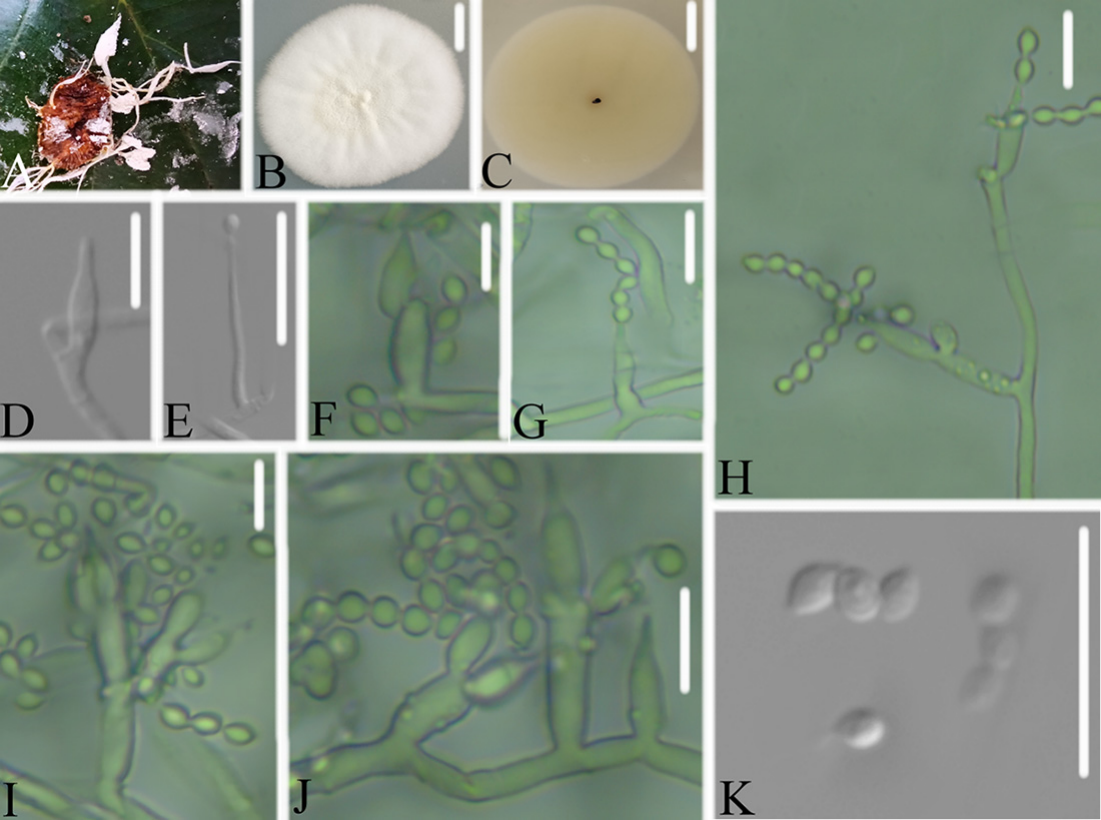
*Samsoniella erucae*. (A) Infected caterpillar (Lepidoptera). (B, C) PDA-containing culture viewed from above (B) and below (C). (D–K). Phialides and conidia. Scale bars: 10 mm (B, C) and 10 μm (D–K).

*Samsoniella erucae* W.H. Chen, Y.F. Han, J.D. Liang & Z.Q. Liang, sp. nov. (MycoBank: 844989) (Fig. 7).

Type: China, Guizhou, Guiyang, Kaiyang County, Monkey-Ear Tiankeng (27°5'12.138” N, 107°0'48.42” E). On a caterpillar (Lepidoptera), 16 November 2020, Wanhao Chen, GZAC KY1112 (holotype), ex-type living cultures, KY11121.

Description: Synnemata arose from different parts of the insect host. Conidiophores were synnematous and arose from the lateral hyphae of the synnemata. Colonies on PDA were 4.6 to 4.8 cm in diameter after 14 d at 25°C, white, comprised of a basal felt hyphal overgrowth, and powdery in the middle during mass sporulation with the reverse light yellowish. Prostrate hyphae were smooth, septate, hyaline, and 1.4 to 1.9 μm in diameter. Erect conidiophores usually arose from the aerial hyphae. Phialides were solitary or in groups of three. Phialides were 6.8 to 13.7 × 1.1 to 1.5 μm with a cylindrical or ellipsoidal basal portion and tapered into a short, distinct neck. Conidia were in chains, hyaline, fusiform to ellipsoidal, one-celled, and 2.3 to 2.9 × 1.1 to 1.5 μm. The sexual state was not observed.

Host: Caterpillar (Lepidoptera).

Locality: Kaiyang County (27°5'12.138” N, 107°0'48.42” E), Guiyang, Guizhou Province, China.

Etymology: Referring to its caterpillar host in the order Lepidoptera.

Additional strain examined: China, Guizhou, Guiyang, Kaiyang County (27°5'12.138” N, 107°0'48.42” E). On a caterpillar (Lepidoptera), 16 November 2020, Wanhao Chen, KY11122.

Remarks: *Samsoniella erucae* was easily identified as *Samsoniella* according to the phylogenetic analysis of the combined data sets (ITS, LSU, RPB2, TEF) (Fig. 1). When comparing the typical characteristics, *S. erucae* was morphologically close to *S. coleopterorum* by its fusiform to ellipsoidal conidia, and it was distinguished from *S. coleopterorum* by its longer phialides (6.8 to 13.7 × 1.1 to 1.5 μm) and its bigger conidia (2.3 to 2.9 × 1.1 to 1.5 μm). *S. erucae* clustered into an independent subclade (Fig. 2) and was distinguished from other species.

**FIG 8:**
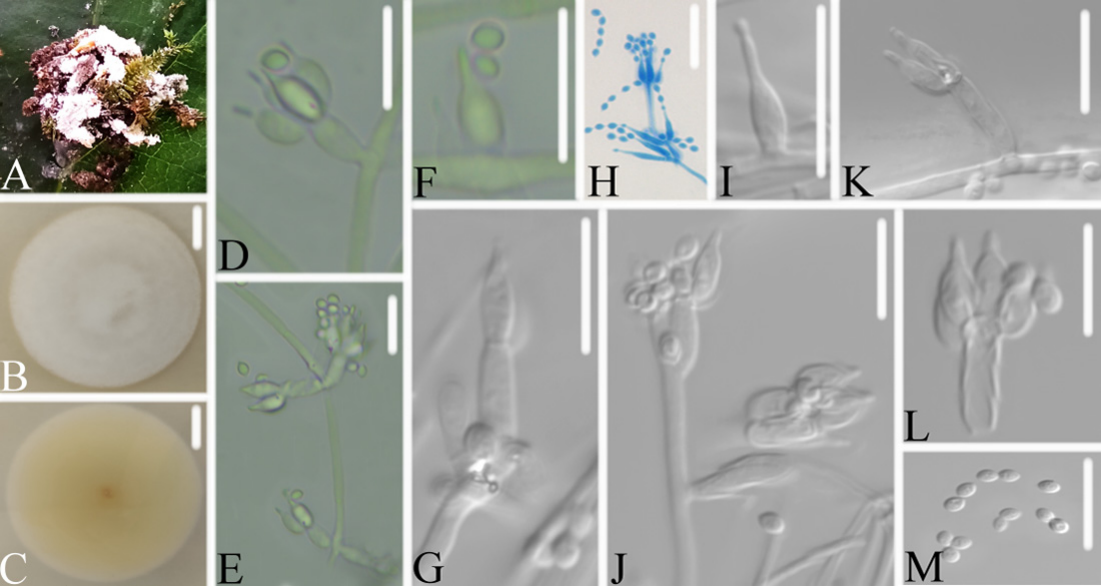
*Samsoniella guizhouensis*. (A) Infected pupa (Lepidoptera). (B, C) PDA-containing culture viewed from above (B) and below (C). (D–M). Phialides and conidia. Scale bars: 10 mm (B, C) and 10 μm (D–M).

*Samsoniella guizhouensis* W.H. Chen, Y.F. Han, J.D. Liang & Z.Q. Liang, sp. nov. (MycoBank: 844990) (Fig. 8).

Type: China, Guizhou, Guiyang, Kaiyang County, Monkey-Ear Tiankeng (27°5'12.138” N, 107°0'48.42” E). On a pupa (Lepidoptera), 16 November 2020, Wanhao Chen, GZAC KY1116 (holotype), ex-type living cultures, KY11161.

Description: Insect pupa was covered completely covered by white mycelium. Conidiophores were mononematous and arose from the lateral hyphae. Colonies on PDA were 4.4 to 4.5 cm in diameter after 14 d at 25°C, white, and comprised of a basal felt and a floccose hyphal overgrowth with the reverse yellowish to pale brown or green. Prostrate hyphae were smooth, septate, hyaline, and 1.3 to 2.5 μm in diameter. Erect conidiophores usually arose from the aerial hyphae. Phialides were solitary or in groups of two. Phialides were 4.9 to 7.9 × 1.7 to 2.1 μm with an ellipsoidal basal portion and tapered into a short, distinct neck. Conidia were in chains, hyaline, fusiform, one-celled, and 2.2 to 2.5 × 1.5 to 1.9 μm. The sexual state was not observed.

Host: Pupa (Lepidoptera).

Locality: Kaiyang County (27°5'12.138” N, 107°0'48.42” E), Guiyang, Guizhou Province, China.

Etymology: Referring to its location in Guizhou Province.

Additional strain examined: China, Guizhou, Guiyang, Kaiyang County (27°5'12.138” N, 107°0'48.42” E). On a pupa (Lepidoptera), 16 November 2020, Wanhao Chen, KY11162.

Remarks: *Samsoniella guizhouensis* was easily identified as *Samsoniella*, according to the phylogenetic analysis of the combined data sets (ITS, LSU, RPB2, TEF) (Fig. 1), and has a close relationship with *S. pupicola* and *S. formicae*. When comparing the typical characteristics, *S. guizhouensis* was easily distinguished from *S. pupicola* and *S. formicae* by its smaller conidia (2.2 to 2.5 × 1.5 to 1.9 μm) and its shorter phialides (4.9 to 7.9 × 1.7 to 2.1 μm).

**FIG 9:**
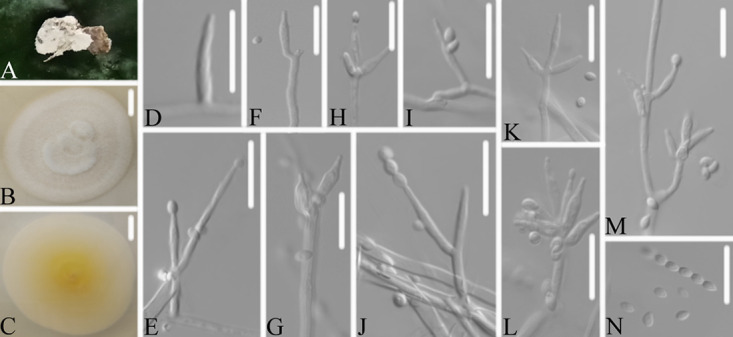
*Samsoniella neopupicola*. (A) Infected pupa (Lepidoptera). (B, C). PDA-containing culture viewed from above (B) and below (C). (D–N) Phialides and conidia. Scale bars: 10 mm (B, C) and 10 μm (D–N).

*Samsoniella neopupicola* W.H. Chen, Y.F. Han, J.D. Liang & Z.Q. Liang, sp. nov. (MycoBank: 844991) (Fig. 9).

Type: China, Guizhou, Guiyang, Kaiyang County, Monkey-Ear Tiankeng (27°5'12.138” N, 107°0'48.42” E). On a pupa (Lepidoptera), 16 November 2020, Wanhao Chen, GZAC KY1132 (holotype), ex-type living cultures, KY11321.

Description: Insect host was completely covered by white mycelium. Conidiophores were mononematous and arose from the lateral hyphae. Colonies on PDA were 5.5 to 5.7 cm in diameter after 14 d at 25°C, white, and comprised of a basal felt and a floccose hyphal overgrowth with the reverse yellowish or pale orange. Prostrate hyphae were smooth, septate, hyaline, and 1.2 to 2.4 μm in diameter. Erect conidiophores usually arose from the aerial hyphae. Phialides were solitary or in groups of five. Phialides were 8.2 to 11.7 × 1.5 to 2.3 μm with a cylindrical basal portion and tapered into a short, distinct neck. Conidia were in chains, hyaline, fusiform, one-celled, and 2.5 to 3.0 × 1.6 to 2.3 μm. The sexual state was not observed.

Host: Pupa (Lepidoptera).

Locality: Kaiyang County (27°5'12.138” N, 107°0'48.42” E), Guiyang, Guizhou Province, China.

Etymology: Referring to its pupa host in the order Lepidoptera.

Additional strain examined: China, Guizhou, Guiyang, Kaiyang County (27°5'12.138” N, 107°0'48.42” E). On a pupa (Lepidoptera), 16 November 2020, Wanhao Chen, KY11322.

Remarks: *Samsoniella neopupicola* was easily identified as *Samsoniella*, according to the phylogenetic analysis of the combined data sets (ITS, LSU, RPB2, TEF) (Fig. 1), and has a close relationship with *S. alboaurantia* and *S. cardinalis*. When comparing the typical characteristics, *S. neopupicola* was easily distinguished from *S. alboaurantia* by its longer phialides (8.2 to 11.7 × 1.5 to 2.3 μm) and its Lepidoptera pupa host. It was distinguished from *S. cardinalis* by its smaller phialides (8.2 to 11.7 × 1.5 to 2.3 μm).

**FIG 10:**
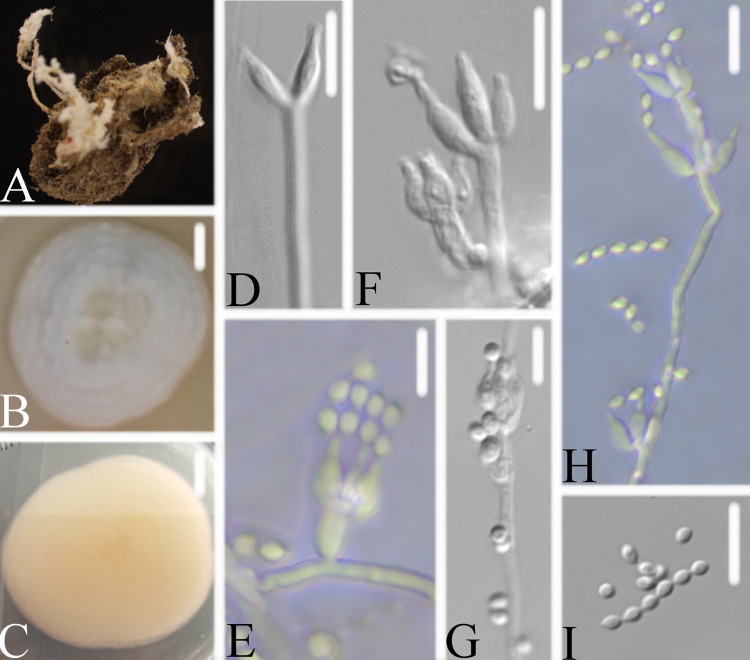
*Samsoniella tiankengensis*. (A) Infected pupa (Lepidoptera). (B, C) PDA-containing culture viewed from above (B) and below (C). (D–I) Phialides and conidia. Scale bars: 10 mm (B, C) and 10 μm (D–I).

*Samsoniella tiankengensis* W.H. Chen, Y.F. Han, J.D. Liang & Z.Q. Liang, sp. nov. (MycoBank: 844992) (Fig. 10).

Type: China, Guizhou, Guiyang, Kaiyang County, Monkey-Ear Tiankeng (27°5'12.138” N, 107°0'48.42” E). On a pupa (Lepidoptera), 16 November 2020, Wanhao Chen, GZAC KY1174 (holotype), ex-type living cultures, KY11741.

Description: Synnemata arose from different parts of the insect pupa. Conidiophores were synnematous and arose from the lateral hyphae of the synnemata. Colonies on PDA were 5.3 to 5.6 cm in diameter after 14 d at 25°C, white to light pink, and comprised of a basal felt and a cottony hyphal overgrowth with the reverse light yellowish. Prostrate hyphae were smooth, septate, hyaline, and 1.5 to 2.5 μm in diameter. Erect conidiophores usually arose from the aerial hyphae. Phialides were solitary or in groups of four. Phialides were 5.4 to 10.4 × 1.3 to 2.2 μm with a cylindrical or subellipsoidal basal portion and tapered into a short, distinct neck. Conidia were in chains, hyaline, ellipsoidal, one-celled, and 2.3 to 2.8 × 1.6 to 1.8 μm. The sexual state was not observed.

Host: Pupa (Lepidoptera).

Locality: Kaiyang County (27°5'12.138” N, 107°0'48.42” E), Guiyang, Guizhou Province, China.

Etymology: Referring to its location in Tiankeng.

Additional strain examined: China, Guizhou, Guiyang, Kaiyang County (27°5'12.138” N, 107°0'48.42” E). On a pupa (Lepidoptera), 16 November 2020, Wanhao Chen, KY11742.

Remarks: *Samsoniella tiankengensis* was easily identified as *Samsoniella* according to the phylogenetic analysis of the combined data sets (ITS, LSU, RPB2, TEF) (Fig. 1). When comparing the typical characteristics, *S. tiankengensis* was morphologically close to *S. erucae* and *S. coleopterorum* by its fusiform or ellipsoidal conidia, and it was distinguished from *S. erucae* and *S. coleopterorum* by its shorter phialides (5.4 to 10.4 × 1.3 to 2.2 μm), its bigger conidia (2.3 to 2.8 × 1.6 to 1.8 μm), and its pupa host. *S. tiankengensis* clustered into an independent subclade (Fig. 2) and was distinguished from other species.

## DISCUSSION

Monkey-Ear Tiankeng is an important modern refugium. It contains cliffs, caves, and an underground river. Following a vertical distribution pattern, the bottom of Tiankeng is the center of the biodiversity, and the species distribution gradually increases from the pit mouth to the pit bottom, such that it can be used as a natural refuge for organisms in rocky karst, desert, and mountainous areas (26, 27). Previous studies have shown that many species of Ascomycota and Basidiomycota are present in the karst cave (an important refugium) (28, 29). Deng and Wu (30) showed that abundant species of Basidiomycota were found in Tiankeng of Leye County. However, few studies have addressed the species diversity of Ascomycota in Tiankeng.

*Cordyceps*-like fungi are important Ascomycota that originated in remote mountains and dense forests and have become closely intertwined with people’s lives (31). *Cordyceps*-like fungi were previously used as traditional medicinal fungi and as a type of health food, and their uses have expanded into other fields, such as biological materials, biochromes, and new bioactive substances, because of the expansion of their members and their host range. The physiological effects of extracts from *Cordyceps*-like fungi and their active components have been involved in promoting the production of cytokines, such as interleukin and tumor necrosis factor-α, and used for their antioxidant, anticancer, hypolipidemic, hypoglycemic, antifatigue, anti-aging, cholesterol-lowering, blood-pressure-lowering, antidepressant, aphrodisiac, and kidney-protecting properties (32). *Cordyceps*-like fungi also have broad application prospects in the fields of fungal feed additives, the environment, nano-materials, and biotransformation (33).

In the present study, the species diversity of a *Cordyceps*-like fungus in Monkey-Ear Tiankeng was investigated. 77 specimens were collected, and 51 strains were isolated. In a combined analysis of ITS sequences and morphological characteristics, 35 strains were identified: Beauveria bassiana (11 strains), *B. pseudobassiana* (5 strains), *Cordyceps tenuipes* (5 strains), *C. fumosorosea* (2 strains), *C. cateniannulata* (2 strains), *C. cateniobliqua* (9 strains), and *C. javanica* (1 strain). A total of 16 strains were clustered into the following genera: *Akanthomyces* (4 strains), *Cordyceps* (2 strains), and *Samsoniellla* (10 strains). More information is needed for further identification. Eight new species: *A. araneosus*, *A. tiankengensis*, *C. tiankengensis*, *S. formicae*, *S. erucae*, *S. guizhouensis*, *S. neopupicola*, and *S. tiankengensis*, were established and described according to a multilocus phylogenetic analysis and their morphological characteristics. Our results showed that *Cordyceps*-like fungi are abundant in Monkey-Ear Tiankeng. Interestingly, the month of the sampling was November, which is not an active period of growth and reproduction for *Cordyceps*-like fungi, revealing that unconventional time sampling should not be ignored, especially for a special eco-environment, such as Tiankeng. However, further research is needed to confirm whether the diversity of the *Cordyceps*-like fungi in Tiankeng, especially for the new species, is related to the sampling time and the environment.

*Cordyceps*-like fungi are all-rounders in their nutrition intake. The nutritional model of *Cordyceps*-like fungi was found to range from plants (including living plants and plant residues) to animals (especially insects) and even to fungi (34). The initial sources of nutrition of the *Cordyceps*-like fungi were plants and their residues. Beauveria bassiana is an important traditional medicinal fungus and was successfully applied in biocontrol. Previous studies have revealed that B. bassiana could cause endophytic colonization in plants, induce systemic resistance against plant pathogens, promote plant growth, and enhance the resistance of plants to insect pests (35, 36). *Cordyceps fumosorosea* is usually used for biocontrol and can enhance plant growth and prevent insect pests (37). Most *Cordyceps*-like fungi are soil-dwelling microbes (38), and they may play an important role in the cycling of carbon and nutrients in their habitats (39). Previous studies have found that plant diversity in the Tiankeng karst region is characterized by rich species diversity, ancient origins, and characteristics of secondary vegetation (3). The relationship among the *Cordyceps*-like fungi, plants, and Tiankeng is worthy of further research.

Tiankeng acts as a refugium for biodiversity amid changing global climates, as has been shown in many studies (10, 17). A previous study showed that some ancient (*Alsophila spinulosa*) and unique plants (cool-adapted plants) are present in Tiankeng (23, 40). Interestingly, abundant *Cordyceps*-like fungi were found in Monkey-Ear Tiankeng, and the hosts of the new species were relatively simple. *Akanthomyces* species are often isolated from soil, insects, and spiders (41, 42), and *Samsoniella* species are often found initiated on a pupa or larva of Lepidoptera, beetles, bees, or ants (43, 44). Furthermore, sampling was performed in November, when the temperature of the environment was lower. Whether these fungi are more ancient than others, have adapted to the cold environment, or have coevolved with their hosts and have special metabolizing processes is worthy of further research.

## MATERIALS AND METHODS

### Specimen collection and identification.

77 infected insect and spider specimens (named KY1101 to KY1177) were collected from Monkey-Ear Tiankeng (27°5'12.138” N, 107°0'48.42” E), Kaiyang County, Guiyang, Guizhou Province, on 16 November 2020. The area belongs to a subtropical monsoon humid climate zone that receives an annual precipitation of 1,141 to 1,547 mm and has an annual average temperature of 10.6 to 15.3°C (26). The isolation of the strains was conducted as described by Chen et al. (45). Fungal colonies emerging from specimens were isolated and cultured at 25°C for 14 days under 12 h light/12 h dark conditions, following the protocol described by Zou et al. (46). The specimens and the isolated strains were deposited in the Institute of Fungus Resources, Guizhou University (formally Herbarium of Guizhou Agricultural College; code: GZAC), Guiyang City, Guizhou, China.

The macroscopic and microscopic morphological characteristics of the fungi were examined, especially for the arrangement, shape, and measurement of phialides and conidia. The growth rates were also determined from potato dextrose agar cultures incubated at 25°C for 14 days. The hyphae and conidiogenous structures were mounted in lactophenol cotton blue or a 20% lactate solution and observed with an optical microscope (OM, DM4 B, Leica, Germany).

### DNA extraction, PCR amplification, and nucleotide sequencing.

DNA extraction was carried out using the Fungal Genomic DNA Extraction Kit (DP2033, BioTeke Corporation) in accordance with Liang et al. (47). The extracted DNA was stored at −20°C. The amplification of the internal transcribed spacer (ITS) region, the large subunit rRNA (LSU) loci, the small subunit rRNA (SSU), the RNA polymerase II largest subunit 2 (RPB2), and the translation elongation factor 1 alpha (TEF) was determined by polymerase chain reaction (PCR) as described by White et al. (48), Castlebury et al. (49), and van den Brink et al. (50), respectively. Primer sequence information is shown in Table S1. The PCR products were purified and sequenced at Sangon Biotech (Shanghai) Co. The resulting sequences were submitted to GenBank (Table 1).

**TABLE 1:** Taxa included in the phylogenetic analyses

Species	Strain no.	GenBank accession no.				Reference
		ITS	LSU	RPB2	TEF	
*Akanthomyces aculeatus*	HUA 186145		MF416520		MF416465	62
*Akanthomyces aculeatus*	TS772	KC519371	KC519370		KC519366	63
*Akanthomyces araneogenum*	GZUIFDX2	KU893153	MH978179	MH978185	MH978187	64
*Akanthomyces araneogenum*	GZUIFDX1	KU893152	MH978178	MH978184		64
*Akanthomyces araneogenum*	GZUIFSN1	MH978177	MH978180	MH978186	MH978188	64
*Akanthomyces araneosus*	KY11341	ON502826	ON502832	ON525442	ON525443	This study
*Akanthomyces araneosus*	KY11342	ON502844	ON502837	ON525444	ON525445	This study
*Akanthomyces attenuatus*	CBS 402.78	AJ292434	AF339565	EF468935	EF468782	65
*Akanthomyces coccidioperitheciata*	NHJ 6709	JN049865	EU369042	EU369086	EU369025	65
*Akanthomyces farinosa*	CBS 541.81	AY624180			JQ425686	66
*Akanthomyces lecanii*	CBS 101247	JN049836	AF339555	DQ522466	DQ522359	65
*Akanthomyces neocoleopterorum*	GY11241	MN093295	MN093296	MN097812	MN097813	41
*Akanthomyces neocoleopterorum*	GY11242	MN093297	MN093298	MN097814	MN097815	41
*Akanthomyces lepidopterorum*	SD05151	MT705971	MT705973	MT727044		52
*Akanthomyces lepidopterorum*	SD05152	MT705972	MT705974	MT727045		52
*Akanthomyces noctuidarum*	BCC 36265	MT356072	MT356084	MT477987	MT477978	42
*Akanthomyces kanyawimiae*	TBRC 7244	MF140752	MF140716		MF140836	67
*Akanthomyces pissodis*	CBS 118231		KM283799	KM283864	KM283822	52
*Akanthomyces pyralidarum*	BCC28816	MT356080	MT356091	MT478007	MT477982	42
*Akanthomyces sulphureus*	TBRC 7248	MF140758	MF140722	MF140812	MF140843	67
*Akanthomyces thailandicus*	TBRC 7245	MF140754		MF140809	MF140839	67
*Akanthomyces tiankengensis*	KY11571	ON502848	ON502825	ON525446	ON525447	This study
*Akanthomyces tiankengensis*	KY11572	ON502821	ON502827	ON525448	ON525449	This study
*Akanthomyces tortricidarum*	BCC72638	MT356076	MT356088	MT477992	MT478004	42
*Akanthomyces tuberculatus*	OSC 111002	JN049830	DQ518767	DQ522435	DQ522338	34
*Akanthomyces waltergamsii*	TBRC 7252	MF140748	MF140714		MF140834	67
*Ascopolyporus polychrous*	P.C. 546		DQ118737		DQ118745	68
*Ascopolyporus villosus*	ARSEF 6355		AY886544		DQ118750	68
Beauveria bassiana	ARSEF 1564	HQ880761		HQ880905	HQ880974	69
*Beauveria brongniartii*	ARSEF 617	HQ880782		HQ880926	HQ880991	69
*Beauveria brongniartii*	BCC 16585	JN049867	JF415967	JF415991	JF416009	65
*Beauveria caledonica*	ARSEF 2567	HQ880817	AF339520	HQ880961	EF469057	63
*Blackwellomyces cardinalis*	OSC 93609		AY184962	DQ522422	DQ522325	63
*Blackwellomyces cardinalis*	OSC 93610	JN049843	AY184963	EF469106	EF469059	65
*Blackwellomyces pseudomilitaris*	NBRC 101409	JN943305	JN941393			70
*Blackwellomyces pseudomilitaris*	NBRC 101410	JN943307	JN941394			70
*Cordyceps amoene-rosea*	CBS 107.73	AY624168	MG665224	MG665234		67
*Cordyceps amoene-rosea*	CBS 729.73	AY624169	MG665225	MG665235	HM161732	67
*Cordyceps bifusispora*	EFCC 5690		EF468806	EF468909	EF468746	51
*Cordyceps bifusispora*	EFCC 8260		EF468807	EF468910	EF468747	51
*Cordyceps cateniannulata*	CBS 152.83	AY624172	MG665226		JQ425687	67
*Cordyceps cateniobliqua*	CBS 153.83	AY624173		MG665236	JQ425688	67
*Cordyceps chiangdaoensis*	TBRC 7274	KT261393	MF140732		KT261403	67
*Cordyceps coleopterorum*	CBS 110.73	AY624177	JF415988	JF416006	JF416028	66
*Cordyceps farinosa*	CBS 111113	AY624181	FJ765253	GU979973	GQ250022	67
*Cordyceps fumosorosea*	CBS 107.10	AY624184	MG665227	MG665237	HM161735	67
*Cordyceps fumosorosea*	CBS 337.52	EF411219	MG665228		MG665233	67
*Cordyceps javanica*	CBS 134.22	AY624186	MG665231		JQ425683	67
*Cordyceps kyusyuensis*	EFCC 5886		EF468813	EF468917	EF468754	51
Cordyceps militaris	OSC 93623	JN049825	AY184966		DQ522332	51
*Cordyceps morakotii*	TBRC 7275	KT261388	MF140730		KT261398	67
*Cordyceps morakotii*	TBRC 7276	KT261390	MF140731		KT261400	67
*Cordyceps ninchukispora*	EFCC 5197		EF468820		EF468760	51
*Cordyceps ninchukispora*	EFCC 5693		EF468821		EF468762	51
*Cordyceps oncoperae*	AFSEF 4358		AF339532	EF468936	EF468785	51
*Cordyceps tiankengensis*	KY11141	ON502831	ON502824		ON525440	This study
*Cordyceps tiankengensis*	KY11142	ON502829	ON502836		ON525441	This study
*Cordyceps piperis*	CBS 116719		AY466442	EU369083	DQ118749	71
*Cordyceps pruinosa*	ARSEF 5413	JN049826	AY184968	DQ522451	DQ522351	65
*Cordyceps tenuipes*	ARSEF 5135	AY624196	JF415980	JF416000	JF416020	65
*Gibellula longispora*	NHJ 12014			EU369075	EU369017	71
*Gibellula pulchra*	NHJ 10808		EU369035	EU369076	EU369018	71
*Gibellula ratticaudata*	ARSEF 1915		DQ518777	DQ522467	DQ522360	71
*Hevansia arachnophila*	NHJ 10469		EU369031		EU369008	71
*Hevansia cinerea*	NHJ 3510			EU369070	EU369009	71
*Hevansia novoguineensis*	NHJ 11923		EU369032	EU369072	EU369013	71
*Leptobacillium coffeanum*	COAD 2057	MF066034	MF066032			72
*Leptobacillium coffeanum*	COAD 2061	MF066035	MF066033			72
*Leptobacillium filiforme*	URM 7918	MH979338	MH979399			73
Purpureocillium lilacinum	CBS 284.36			EF468941	EF468792	65
Purpureocillium lilacinum	CBS 431.87	HQ842812	EF468844	EF468940	EF468791	51
*Samsoniella alboaurantium*	CBS 240.32		JF415979	JF415999	JF416019	65
*Samsoniella alboaurantium*	CBS 262.58		AB080087	MF416448	MF416497	67
*Samsoniella alpina*	YFCC 5818		MN576809	MN576923	MN576979	74
*Samsoniella alpina*	YFCC 5831		MN576810	MN576924	MN576980	74
*Samsoniella antleroides*	YFCC 6016		MN576803	MN576917	MN576973	74
*Samsoniella antleroides*	YFCC 6113		MN576804	MN576918	MN576974	74
*Samsoniella aurantia*	TBRC 7271		MF140728	MF140818	MF140846	67
*Samsoniella aurantia*	TBRC 7272		MF140727	MF140817	MF140845	67
*Samsoniella cardinalis*	YFCC 5830		MN576788	MN576902	MN576958	74
*Samsoniella cardinalis*	YFCC 6144		MN576786	MN576900	MN576956	74
*Samsoniella coleopterorum*	A19501	MT626376		MN101585	MN101586	43
*Samsoniella coleopterorum*	A19502	MT626625		MN101587	MT642602	43
*Samsoniella cristata*	YFCC 6021		MN576791	MN576905	MN576961	74
*Samsoniella cristata*	YFCC 6023		MN576792	MN576906	MN576962	74
*Samsoniella erucae*	KY11121	ON502828	ON502835	ON525424	ON525425	This study
*Samsoniella erucae*	KY11122	ON502847	ON502822	ON525426	ON525427	This study
*Samsoniella formicae*	KY11041	ON502852		ON525420	ON525421	This study
*Samsoniella formicae*	KY11042	ON502842		ON525422	ON525423	This study
*Samsoniella guizhouensis*	KY11161	ON502823	ON502830	ON525428	ON525429	This study
*Samsoniella guizhouensis*	KY11162	ON502845	ON502846	ON525430	ON525431	This study
*Samsoniella hepiali*	ICMM 82-2		MN576794	MN576908	MN576964	74
*Samsoniella hepiali*	YFCC 661		MN576795	MN576909	MN576965	74
*Samsoniella hymenopterorum*	A19521	MN128224		MT642604	MN101588	43
*Samsoniella hymenopterorum*	A19522	MN128081		MN101590	MN101591	43
*Samsoniella inthanonensis*	TBRC 7915	MF140761		MF140815	MF140849	67
*Samsoniella inthanonensis*	TBRC 7916	MF140760		MF140814	MF140848	67
*Samsoniella kunmingensis*	YHH 16002		MN576802	MN576916	MN576972	74
*Samsoniella lanmaoa*	YFCC 6148		MN576789	MN576903	MN576959	74
*Samsoniella lanmaoa*	YFCC 6193		MN576790	MN576904	MN576960	74
*Samsoniella lepidopterorum*	DL10071	MN128076		MN101593	MN101594	43
*Samsoniella lepidopterorum*	DL10072	MN128084		MT642605	MT642606	43
*Samsoniella neopupicola*	KY11321	ON502843	ON502839	ON525432	ON525433	This study
*Samsoniella neopupicola*	KY11322	ON502834	ON502833	ON525434	ON525435	This study
*Samsoniella pseudogunii*	GY407201	MZ827470	MZ827010	MZ855239	MZ855233	44
*Samsoniella pseudogunii*	GY407202	MZ831863	MZ831865	MZ855240	MZ855234	44
*Samsoniella pupicola*	DY101681	MZ827085	MZ827009	MZ855237	MZ855231	44
*Samsoniella pupicola*	DY101682	MZ827008	MZ827635	MZ855238	MZ855232	44
*Samsoniella ramose*	YFCC 6020		MN576805	MN576919	MN576975	74
*Samsoniella tiankengensis*	KY11741	ON502840	ON502838	ON525436	ON525437	This study
*Samsoniella tiankengensis*	KY11742	ON502849	ON502841	ON525438	ON525439	This study
*Samsoniella tortricidae*	YFCC 6013		MN576807	MN576921	MN576977	74
*Samsoniella tortricidae*	YFCC 6131		MN576806	MN576920	MN576976	74
*Samsoniella yunnanensis*	YFCC 1527		MN576812	MN576926	MN576982	74
*Samsoniella yunnanensis*	YFCC 1824		MN576813	MN576927	MN576983	74
*Simplicillium formicae*	MFLUCC 18–1379	MK766511	MK766512		MK926451	75
*Simplicillium lamellicola*	CBS 116.25	AJ292393	AF339552	DQ522462	DQ522356	51
*Simplicillium lanosoniveum*	CBS 101267	AJ292395	AF339554	DQ522463	DQ522357	51
*Simplicillium lanosoniveum*	CBS 704.86		AF339553	DQ522464	DQ522358	51
*Simplicillium obclavatum*	CBS 311.74		AF339517		EF468798	51

### Sequence alignment and phylogenetic analyses.

Lasergene software (version 6.0, DNASTAR) was applied for the assembly and editing of the DNA sequences in this study. ITS, LSU, RPB2, and TEF sequences were downloaded from GenBank, based on Sung et al. (51), Chen et al. (41, 43, 44, 52), Li et al. (53), Aini et al. (42), and others selected on the basis of Basic Local Alignment Search Tool (BLAST) algorithm-based searches in GenBank (Table 1). A single-locus data set was aligned and edited using multiple alignment using fast Fourier transform (MAFFT) v7.037b (54) and Molecular Evolutionary Genetics Analysis version 6 (55). Combined sequences of ITS, LSU, RPB2, and TEF were performed in SequenceMatrix v.1.7.8 (56). The model for the Bayesian analysis was selected using ModelFinder (57) with PhyloSuite software (58).

The combined loci were analyzed using Bayesian inference (BI) and maximum likelihood (ML) methods. For the BI, a Markov chain Monte Carlo (MCMC) algorithm was used to generate phylogenetic trees with Bayesian probabilities for the combined sequence data sets using MrBayes v.3.2 (59). The Bayesian analysis resulted in 20,001 trees after 10,000,000 generations. The first 4,000 trees, representing the burn-in phase of the analyses, were discarded, while the remaining 16,001 trees were used to calculate the posterior probabilities for the majority rule consensus tree. After the analysis was finished, each run was examined using the Tracer v1.5 program (60) to determine the burn-in and to confirm that both runs had converged. The ML analysis was designed with IQ-TREE (61), and the model was automatically selected by the software.
