# Supplementary material for: Species Diversity of Cordyceps-Like Fungi in the Tiankeng Karst Region of China
Source: Microbiol Spectr. 2022 Sep 12;10(5):e01975-22. doi: 10.1128/spectrum.01975-22 (PMC9603550; doi:10.1128/spectrum.01975-22)
Supplement: Supplemental file 1 — Table S1. Download spectrum.01975-22-s0001.pdf, PDF file, 0.3 MB [file spectrum.01975-22-s0001.pdf]

Table S1. Primers information for 5 DNA sequences

Name	Length	Direction	Sequence 5´-3´
ITS5	22	forward	GGAAGTAAAAGTCGTAACAAGG
ITS4	20	reverse	TCCTCCGCTTATTGATATGC
NS1	19	forward	GTAGTCATATGCTTGTCT C
NS4	20	reverse	CTTCCGTCAATTCCTTTAAG
LROR	17	forward	ACCCGCTGAACTTAAGC
LR5	17	reverse	TCCTGAGGGAAACTTCG
CRPB1	20	forward	CAYCCWGGYTTYATCAAGAA
RPB1Cr	23	reverse	CCNGCDATNTCRTTRTCCATRTA
RPB2-5F3	20	forward	GACGACCGTGATCACTTTGG
RPB2-7Cr2	20	reverse	CCCATGGCCTGTTTGCCCAT
983F	23	forward	GCYCCYGGHCAYCGTGAYTTYAT
2218R	23	reverse	ATGACACCRACRGCRACRGTYTG
